# Child with Testicular Pain

**DOI:** 10.5811/cpcem.2017.11.36530

**Published:** 2018-01-11

**Authors:** Nicholas Otts, Ryan L. Webb, Josh Greenstein, Barry Hahn

**Affiliations:** * Staten Island University Hospital, Northwell Health, Department of Emergency Medicine, Staten Island, New York; † Staten Island University Hospital, Northwell Health, Department of Radiology, Staten Island, New York

## CASE PRESENTATION

**Image 2:**
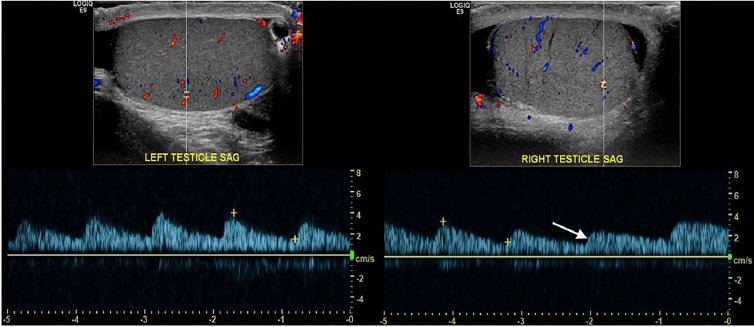
Transverse ultrasound image of the left testicle obtained with a 15Mhz linear-array transducer using spectral Doppler demonstrates normal arterial wave form and flow (2A). Spectral Doppler of the right testicle (white arrow) demonstrates comparatively decreased systolic velocities with slowing of the upstroke (2B).

An 11-year-old boy presented to the emergency department (ED) with sudden onset severe atraumatic right testicular pain, associated with nausea and vomiting. On examination, the patient exhibited a slight horizontal lie of the right testicle as well as an absent cremasteric reflex on the right. Urology was emergently consulted. Though vascular flow was noted bilaterally on spectral Doppler (Images 2), the patient underwent surgical detorsion due to the whirlpool sign seen on point-of-care ultrasound (POCUS) of spermatic cord (Image).

## DIAGNOSIS

Testicular torsion with whirlpool sign: Testicular torsion occurs when twisting of the spermatic cord results in a subsequent loss of blood supply to the ipsilateral testicle. Testicular torsion is primarily a pediatric problem, with a bimodal age distribution of the first year of life and early adolescence. Evaluation of testicular flow by color and spectral Doppler is the primary method of diagnosis.^1^ However, evaluation with Doppler can be non-diagnostic due to the presence of low-velocity blood flow. Direct visualization of the spermatic cord by POCUS may elicit the whirlpool sign or an echogenic mass twisted around a central axis. As demonstrated by this case, whirlpool sign is diagnostic of torsion and can help identify torsion when Doppler is non-diagnostic.^2^,^3^ Thus, the whirlpool sign, although not frequently mentioned in emergency medicine literature, can be critical to the diagnosis of torsion in the ED and should be an ultrasound finding known to all emergency physicians.

CPC-EM Capsule

What do we already know about this clinical entity?

The whirlpool sign is an important diagnostic marker in the evaluation of suspected testicular torsion by point- of-care ultrasound.

What is the major impact of the image(s)?

The whirlpool sign is a characteristic sonographic finding indicative of testicular torsion.

How might this improve emergency medicine practice?

Visualization of the whirlpool sign allows confident diagnosis of testicular torsion.

## Figures and Tables

**Image 1:**
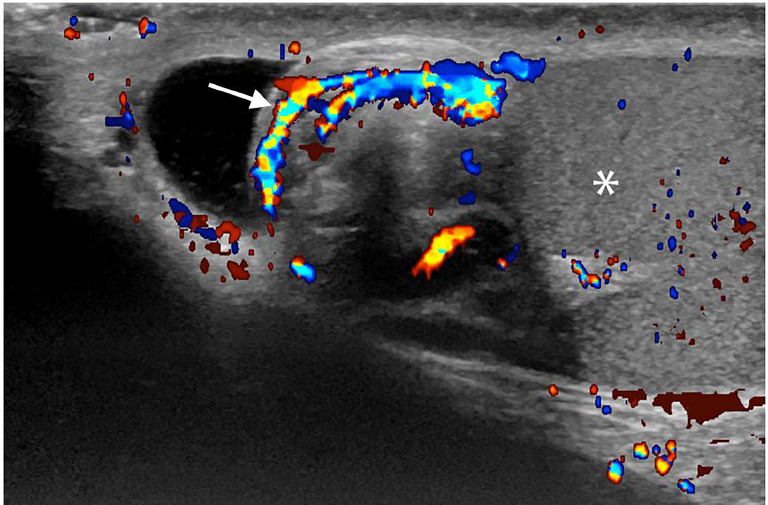
Ultrasound of the spermatic cord demonstrates an edematous, heterogeneous extra testicular mass representing the twisted spermatic cord, with whorled color flow, the “whirlpool sign”(white arrow) around a central axis. Immediately adjacent is the testicle (white asterisk).
